# Integrating ChatGPT and Escape Room with a Geriatric 5Ms Curriculum for Interprofessional Residents: A Pilot

**DOI:** 10.1093/geroni/igaf122.3269

**Published:** 2025-12-31

**Authors:** Huai Cheng, Erica Allen, Jordan Risher, Kimberly Heckmann, Antonio Valenzuela, Jennifer Schutz, Robin Rabey, Edward Rattner

**Affiliations:** Minneapolis VA Hospital, Minneapolis, Minnesota, United States; Minneapolis VA Hospital, Minneapolis, Minnesota, United States; Minneapolis VA Hospital, Minneapolis, Minnesota, United States; Minneapolis VA Hospital, Minneapolis, Minnesota, United States; Minneapolis VA Hospital, Minneapolis, Minnesota, United States; Minneapolis VA Hospital, Minneapolis, Minnesota, United States; Minneapolis VA Hospital, Minneapolis, Minnesota, United States; Minneapolis VA Hospital, Minneapolis, Minnesota, United States

## Abstract

Geriatric 5Ms are core components of age-friendly health system (AFHS). Awareness and knowledge of geriatric 5Ms and AFHS among health professional trainees are low. ChatGPT application to geriatrics education is less studied. With growingly ChatGPT application to medical education, we developed a 2-hour case-based interprofessional geriatric 5M curriculum integrated with ChatGPT as an interaction tool and used the Escape Room (ER) as outcome assessment at a VA medical center. This curriculum aims to demonstrate the application of Geriatrics 5Ms and ChatGPT to the care of older adults for Geri-pharmacy, Nurse practitioners (NP) and Internal medicine (IM) residents (N = 6). After a brief didactic introduction to geriatric 5Ms, AFHS, and interprofessional teamwork approach the case was then unfolded with 7 geriatric 5Ms questions, Responses to 7 questions by the trainees versus and ChatGPT were compared and discussed by the trainees (n = 6), pharmacy, NP and geriatrics faculty (n = 4) and simulation center staff (n = 3). All residents attended a 20-minute orientation to ER, they were locked in ER which was designed to assess whether they can efficiently and effectively apply the geriatric 5Ms knowledge to the case of an older patient ready to be discharged from hospital to home. The residents successfully escaped ER as a team in 22 minutes, which was followed by a 15-minute debriefing. This pilot curriculum was simple to implement. Trainee engagement in geriatrics was enhanced using novel ChatGPT and the Escape Room. Further testing and evaluation of this curriculum is planned.

